# Draft Genome Sequences of Two *Thiomicrospira* Strains Isolated from the Brine-Seawater Interface of Kebrit Deep in the Red Sea

**DOI:** 10.1128/genomeA.00110-16

**Published:** 2016-03-10

**Authors:** Guishan Zhang, Mohamed Fauzi Haroon, Ruifu Zhang, Tyas Hikmawan, Ulrich Stingl

**Affiliations:** a King Abdullah University of Science and Technology (KAUST), Red Sea Research Center, Thuwal, Saudi Arabia; b Ministry of Agriculture, Institute of Agricultural Resources and Regional Planning, Key Laboratory of Microbial Resources Collection and Preservation, Chinese Academy of Agricultural Sciences, Beijing, People’s Republic of China

## Abstract

Two *Thiomicrospira* strains, WB1 and XS5, were isolated from the Kebrit Deep brine-seawater interface in the Red Sea, Saudi Arabia. Here, we present the draft genome sequences of these gammaproteobacteria*,* which both produce sulfuric acid from thiosulfate in culture.

## GENOME ANNOUNCEMENT

The genus *Thiomicrospira* (*Gammaproteobacteria*, *Thiotrichales*, *Piscirickettsiaceae*) was first proposed by Kuenen and Veldkamp (1) to accommodate one marine bacterial species, *Thiomicrospira pelophila*. At the time of this writing, 11 *Thiomicrospira* species have validly been described, which have been isolated from seawater, intertidal mud flats, coastal sediments, deep-sea hydrothermal fumarole (2), and hypersaline lakes (3). *Thiomicrospira* is widespread in marine environments and plays an important role in the sulfur cycle (4). To date, 18 genome sequences of *Thiomicrospira* strains are available in NCBI GenBank.

Here, we present the genome sequences of *Thiomicrospira* sp. strains WB1 and XS5, which were isolated from the Kebrit Deep brine-seawater interface (24°44′N, 36°17′E) in the Red Sea at a depth of 1,465 m. Phylogenetic analysis of the 16S rRNA genes indicated that strains WB1 and XS5 are most closely related to *Thiomicrospira halophila* (96.53%) and *Thiomicrospira thermophila* (97.28%), respectively.

Strains WB1 and XS5 were enriched and purified at 28°C using a sulfur-oxidizing-bacteria (SOB) medium, which includes the following ingredients: (NH_4_)_2_SO_4_ (1.0 g/liter), MgSO_4_·7H_2_O (1.0 g/liter), CaCl_2_·2H_2_O (0.3 g/liter), KCl (0.6 g/liter), and NaCl (15% [wt/vol]). After autoclaving, the medium was supplemented to contain 10 mM Na_2_S_2_O_3_, 0.5 g/liter K_2_HPO_4_ 5 mM NH_4_Cl, 5 mM EDTA, and 10 mM NaHCO_3_. Bromothymol purple was added as pH indicator at a concentration of 4 mg/liter, and the final pH was adjusted to 7.5 to 8.0 with 1 M HCl and 1 M NaOH. Both strains are aerobic, Gram-negative, flagellated, and capable of growth at up to 20% (wt/vol NaCl) salinity.

Genomic DNA was extracted from the cultured cells using an alkaline lysis method (5) and subsequently sequenced on the Illumina HiSeq 2000 platform. The raw reads were filtered and trimmed using PRINSEQ (version 0.20.4) (6). SOAP*denovo* (version 1.05) (7, 8), with default parameters, was used to assemble the trimmed reads. The assemblies were manually checked and scaffolded based on read mapping. The genome completeness (100%) was assessed using CheckM (version 1.0.3) (9). Protein-coding open reading frames were predicted by Glimmer (version 3.02) (10). rRNAs were predicted by RNAmmer (version 1.2) (11), and tRNAs were predicted by tRNAscan-SE (version 1.21) (12).

The genome of WB1, as presented here, is composed of 6 contigs, with a total length of 2,279,450 bp (*N*_50_, 568,675 kbp) and a G+C content of 53.73%. It contains 2,072 protein-coding genes, 43 tRNAs, and 3 rRNAs. For strain XS5, the genome is composed of 23 contigs, with a total length of 2,633,068 bp (*N*_50_, 2,522,699 bp) and a G+C content of 50.12%. It contains 2,432 protein-coding genes, 45 tRNAs, and 6 rRNAs. Functional annotation by RAST (13) showed the presence of the gene for the osmolarity sensor protein EnvZ and genes related to thiosulfate and sulfur metabolism, supporting the high-salinity adaptation and observed sulfuric acid production during culturing.

### Nucleotide sequence accession numbers.

This whole-genome shotgun project has been deposited at DDBJ/EMBL/GenBank under the accession numbers LQBN00000000 for WB1 and LQBO00000000 for XS5.
